# Multifactor Prediction of Embryo Transfer Outcomes Based on a Machine Learning Algorithm

**DOI:** 10.3389/fendo.2021.745039

**Published:** 2021-11-02

**Authors:** Ran Liu, Shun Bai, Xiaohua Jiang, Lihua Luo, Xianhong Tong, Shengxia Zheng, Ying Wang, Bo Xu

**Affiliations:** ^1^Reproductive and Genetic Hospital, The First Affiliated Hospital of USTC, Division of Life Sciences and Medicine, University of Science and Technology of China, Hefei, China; ^2^Department of Neurosurgery, The First Affiliated Hospital of USTC, Division of Life Sciences and Medicine, University of Science and Technology of China, Hefei, China

**Keywords:** machine learning, embryo transfer, hormone replacement cycle, good-quality embryo ratio, endometrial thickness

## Abstract

*In vitro* fertilization-embryo transfer (IVF-ET) technology make it possible for infertile couples to conceive a baby successfully. Nevertheless, IVF-ET does not guarantee success. Frozen embryo transfer (FET) is an important supplement to IVF-ET. Many factors are correlated with the outcome of FET which is unpredictable. Machine learning is a field of study that predict various outcomes by defining data attributes and using relevant data and calculation algorithms. Machine learning algorithm has been widely used in clinical research. The present study focuses on making predictions of early pregnancy outcomes in FET through clinical characters, including age, body mass index (BMI), endometrial thickness (EMT) on the day of progesterone treatment, good-quality embryo rate (GQR), and type of infertility (primary or secondary), serum estradiol level (E2) on the day of embryo transfer, and serum progesterone level (P) on the day of embryo transfer. We applied four representative machine learning algorithms, including logistic regression (LR), conditional inference tree, random forest (RF) and support vector machine (SVM) to build prediction models and identify the predictive factors. We found no significant difference among the models in the sensitivity, specificity, positive predictive rate, negative predictive rate or accuracy in predicting the pregnancy outcome of FET. For example, the positive/negative predictive rate of the SVM (gamma = 1, cost = 100, 10-fold cross validation) is 0.56 and 0.55. This approach could provide a reference for couples considering FET. The prediction accuracy of the present study is limited, which suggests that there may be some other more effective predictors to be developed in future work.

## Introduction

Frozen embryo transfer (FET) can not only avoid the occurrence of ovarian hyperstimulation syndrome but also avoid the adverse effects of superphysiological estrogen and early elevated progesterone on embryo implantation (1). However, it is difficult to predict the success rate objectively during the FET cycle (2). Failure or abortion of embryo transfer (ET) places psychological and economic burdens on the couple. Early knowledge of the outcome can relieve infertile couples from experiencing serious psychological stress and help them develop more reasonable expectations (3). Therefore, identifying factors that can accurately predict the success rate would be clinically significant.

A large number of studies have found that endometrial receptivity is one of the main factors affecting the pregnancy outcome. Appropriate estrogen and progesterone levels and their periodic changes are the key factors that regulate the receptive state of the endometrium. Endometrial thickness (EMT) and morphology are also closely related to endometrial receptivity (4). Yuval found that EMT > 7 mm was one of the necessary conditions for successful pregnancy (5). In addition, type A endometrium has a higher clinical pregnancy rate than type B and C endometrium (6). However, Golbasi et al. did not demonstrate any significant relationship between EMT changes and clinical pregnancy rates during FET cycles (7). The endometrial morphology is subjectively judged by a sonographer, so the comparability is poor. Some clinicians do not believe that endometrial morphology should be used as an index to evaluate endometrial receptivity. In addition, the quality of the embryo and maternal age are also major factors affecting pregnancy outcomes. Until now, it is still uncertain which factors have the best ability to predict pregnancy outcomes during hormone replacement FET cycles.

Machine learning algorithms have been applied in the field of assisted reproduction. Liu et al. established and compared six classification models that can accurately predict early pregnancy loss. They also found that the random forest (RF) model has the highest predictive ability. However, the patients in the study underwent fresh ET, and the predicted result was early pregnancy loss (8). Xi et al. proposed that artificial intelligence (AI) based on determinant-weighting analysis could provide an individualized embryo selection strategy for any given patient and predict the clinical pregnancy rate and twin risk (9). However, there are few studies using clinical characters on the day of ET to predict the early pregnancy outcomes of patients undergoing hormone replacement FET cycles through machine learning algorithms.

We applied four machine learning algorithms, including logistic regression (LR), conditional inference tree, RF, and support vector machine (SVM), to select features and establish prediction models. Finally, we compared above models to predict the outcome of FET (i.e., success or failure of a clinical pregnancy).

## Materials and Methods

### Data Sources

In this study, 401 patients who underwent hormone replacement FET cycles were enrolled in the First Affiliated Hospital of University of Science and Technology of China (USTC) from December 2019 to August 2020. Data included age (the day when oocytes were picked up), body mass index (BMI), type of infertility (primary or secondary), endometrial preparation protocol (gonadotropin releasing hormone-agonist down regulation or non gonadotropin releasing hormone-agonist down regulation), type of transferred embryo (cleaved embryo or blastocyst), number of transferred embryos per transferred cycle, number of good-quality embryos per transferred cycle, good-quality embryo rate (GQR), serum estrogen level (E2) on the day of ET, serum progesterone level (P) on the day of ET, EMT and endometrial morphology (type A or type B) on the day of progesterone treatment. The interval between the day of FET and oocyte picking-up was no more than six months. The clinical characters of the two groups are shown in **Table 1**.

**Table 1:** The clinical characters of the patients.

Clinical characteristics	Total (n=401)	clinical pregnancy (n=204)	Non-clinical pregnancy (n=197)	P
Age (year), mean ± s.d.	31.7 ± 5.3	30.7 ± 4.8	32.7 ± 5.6	0.0002
BMI (kg/m^2^), mean ± s.d.	22.6 ± 3.0	22.7 ± 3.4	22.4 ± 2.6	0.37
Type of infertility, n (%)				0.01
Primary	205 (51.1)	117 (57.4)	88 (44.7)	
Secondary	196 (48.9)	87 (42.7)	109 (55.3)	
Endometrial preparation protocol, n (%)				0.19
GnRH-a-HRT	116 (28.9)	65 (31.9)	51 (25.9)	
HRT	285 (71.1)	139 (68.1)	146 (74.1)	
Number of transferred embryos, n (%)				0.47
1	184 (45.9)	90 (44.1)	94 (47.7)	
2	217 (54.1)	114 (55.9)	103 (52.3)	
Number of good quality embryos per transferred cycle, n (%)				0.0008
0	35 (8.7)	8 (3.9)	27 (13.7)	
1	199 (49.6)	100 (49.0)	100 (50.8)	
2	167 (41.7)	96 (47.1)	70 (36.5)	
good-quality embryo rate (%)	87.8 ± 0.3	93.4 ± 0.2	82.0 ± 0.4	0.0001
Types of embryos transferred, n (%)				0.64
Cleavage embryos	190 (47.4)	99 (48.5)	91 (46.2)	
Blastocysts	211 (52.6)	105 (51.5)	106 (53.8)	
Serum estradiol level on the day of embryo transfer (pg/ml), mean ± s.d.	434.8 ± 444.5	393.9 ± 385.6	477.1 ± 495.6	0.12
Serum progesterone level on the day of embryo transfer (ng/ml), mean ± s.d.	15.8 ± 7.6	15.8 ± 7.4	15.8 ± 7.8	0.95
Endometrial thickness (mm), mean ± s.d.	9.3 ± 1.7	9.5 ± 1.7	9.0 ± 1.6	0.001
Endometrial morphology, n (%)				0.47
Type A	345 (86.0)	178 (87.3)	167 (84.8)	
Type B	56 (14.0)	26 (12.8)	30 (15.2)	

Categorical variables were described as frequencies (percentages), and continuous variables were described as the mean ± standard deviation (SD). Pearson’s chi-square test and Student’s t-test were used for parametric comparisons. P value < 0.05 was considered to indicate statistical significance.

BMI represents body mass index; GnRH-a-HRT represents gonadotropin releasing hormone agonist down-regulation in combination with hormone replacement therapy; HRT represents conventional hormone replacement therapy.

### Endometrial Preparation and ET

From the second to third day of the menstrual period or withdrawal bleeding, the patients were given oral estradiol valerate 6 mg/day. After 8-12 days, according to the vaginal B-ultrasound monitoring of EMT, the hormone regimen was appropriately adjusted. If the endometrial growth was not ideal, we then added progesterone/estradiol (Femoston) red tablets containing 1 mg vaginal medication. When the EMT of the patients was greater than or equal to 8 mm, progesterone was added to transform the endometrium. For patients with day 3 cleavage stage embryos, ET was performed on the fourth day after progesterone was added. For patients with blastocysts, ET was performed on the sixth day after progesterone was added. The same doses of estrogen and progesterone were continued until obtaining a serum β- human chorionic gonadotropin assay 14 or 12 days after ET. If the pregnancy test was positive, hormone replacement continued for another 8 weeks, and the patients were followed with serial ultrasonography to determine fetal viability.

### Endometrial Morphology

The endometrium was divided into three types according to Gonen classification criteria (10). Type A: typical trilinear or multilayered endometrium with strong gyrus in the outer and central layers. The area between the outer layer and the midline of uterine cavity is hypoechoic or anechoic. Type B: homogeneous moderate echo, strong echo in uterine cavity and unclear midline. Type C: homogeneous hyperechoic without midline echo.

### Embryo Score

Cleaved embryo score: The cleaved embryos were graded according to the number and shape of blastomeres, cytoplasmic granules and cytoplasmic fragments. The classification criteria are mainly based on Istanbul consensus (11). Grade I: the size of the blastomeres is uniform, the shape is regular, and the fragments are less than 10%; Grade II: the blastomeres are slightly uneven or irregular, the cytoplasm has granules, and the fragments are between 10% and 20%; Grade III: the blastomeres are obviously uneven or irregular, the cytoplasm has granules, and the fragments are between 20% and 50%; Grade IV: the blastomeres are seriously uneven or irregular, and the cytoplasm has serious granule phenomenon, debris > 50%. Grade I and II embryos with 6-10 blastomeres on day 3 were defined as good-quality embryos.

Blastocyst score: The blastocysts were graded according to Gardner’s blastocyst grading method (12). First, according to the expansion and hatching degree of the blastocysts, the blastocysts were divided into 1-6 grades: Grade 1, early blastocyst, the volume of the blastocyst cavity is less than half of the total volume of the blastocyst; Grade 2, the volume of the blastocyst cavity is more than half of the total volume of the blastocyst; Grade 3, completely expanded blastocyst, the blastocyst cavity occupies the entire blastocyst; Grade 4, after expansion, the volume of the blastocyst cavity is significantly larger than that of the early blastocyst, and the zona pellucida is thinner; Grade 5, the blastocysts hatched from the zona pellucida with lacerations; Grade 6, the blastocysts hatched completely out of the zona pellucida. Grade 3-6 blastocysts need to be scored for their inner cell mass and trophoblast cells. Inner cell mass (ICM) score: Grade A, the number of cells is large, and there are cells around the blastocyst; Grade B, the number of cells is small, and the combination is loose; Grade C, the number of cells is very small. Trophoblast ectoderm (TE) score: Grade A, with more cells and cells distributed around the blastocyst; Grade B, with fewer cells and loose epithelial cells; Grade C, with few cells. Blastocysts with day 5 scores ≥ 3AA, 3AB, 3BA, 3BB or day 6-7 scores ≥ 4AA, 4AB, 4BA, and 4BB were regarded as good-quality blastocysts and cryopreserved as embryos. The embryos transferred included cleavage embryos and blastocysts without preimplantation genetic testing (PGT).

### Evaluation Index

The main outcome was clinical pregnancy. The success of clinical pregnancy was defined as a gestational sac and an active fetal heartbeat on transvaginal ultrasound 4-5 weeks after ET.

### The Relationship Between Clinical Pregnancy and Other Variables

To explore the importance of different factors in the models, we analyzed the correlations among the parameters (Pearson correlation), including age, BMI, E2, P, EMT and GQR. We compared the relationship between the successful group and the failed group.

### Model Establishment

Age, BMI, EMT, GQR, E2, P and type of infertility were chosen as features and clinical pregnancy was used as a prediction result. We used four machine learning techniques, including LR, conditional inference tree, RF, and SVM, to develop fast and automated prediction models. All of the algorithms were implemented in the R (x64 4.0.4) language. In this study, 70% of the samples were randomly selected as the training set, and the remaining 30% were selected as the test set. Brief introductions to each classifier are given below.

### Logistic Regression (LR)

Logistic regression (LR) is a generalized linear model that can predict binary output according to a group of numerical variables. The basic function glm() in R language can be used to fit the LR model. The probability transformation formula of LR is as follows:


$$p=\frac{1}{1+e^{-(\theta_{0}+\theta_{1}X_{1}+\theta_{2}X_{2}+\cdots +\theta_{n}Xn)}}$$

Xi is the eigenvalue, *θ*_i_ is the regression efficiency, and P is the probability. When P ≥ 0.5 and P < 0.5, they are classified into two different categories. LR is the most effective linear classification model and it is simple, intuitive and interpretable. It is the most commonly used model in clinical data analysis.

First, the results of success or failure were used as response variables, and clinical features were used as prediction variables. The training set data were used to construct the LR model, and the coefficients in the model were given. The model based on the training set was used to classify the data of the test set and the logarithmic probability of success was output. Finally, a cross table was used to compare the prediction with the actual situation.

### Support Vector Machine (SVM)

In machine learning, support vector machine (SVM) is one of the methods with a complete theory and good classification effect. SVM has strong generalization ability in small sample size, nonlinear, and high-dimensional pattern recognition problems, and good application prospects in the medical field.

The tune.svm () function in R language set a candidate range for each parameter. A more efficient model was generated and the performance of each parameter combination was output. Eight different gamma (from 0.000001 to 10) and 21 cost parameters (from 0.01 to 1010) were tried. In general, 168 (8 x 21) models were fitted and the results were compared. Based on this parameter combination, we use a new SVM model to predict the sample units of the test set and give the number of errors to select the best parameter combination. Finally, the best parameter combination model is used to classify the test set. The logarithmic probability of success is output. Finally, a cross table is used to compare the prediction with the actual situation.

SVM is a very popular model which has a wide range of applications. It can be applied to the problem that the number of variables is far more than the number of sample units. This kind of problem is very common in the biomedical industry. However, one of the disadvantages of SVM is that it is difficult to understand and express the classification criteria. It is essentially a black box, and it is not as good as RF in modeling when there are many samples.

### Decision Tree (DT)

Decision tree (DT) is a basic classification method. The DT model is a tree structure, which presents the process instances that are classified based on features. It includes traditional decision trees and conditional inference trees. Constructing a DT involves three main steps: feature selection, decision tree generation and decision tree pruning. 1) Feature selection is mainly determined by the degree of information change (i.e., information gain). 2) The generation of a decision tree refers to the classification of purity and homogeneity. The rpart() function in R language can be used to construct a decision tree. 3) Pruning: the purpose is to make the tree simpler to achieve better generalization ability. The prune() function in R language can be used to prune the decision tree.

The conditional inference tree is a variant of the traditional inference tree. The data are divided into two groups according to a variable. The permutation test is used to calculate the P-value of the two groups, and the variable with the minimum P-value is selected as the grouping node. This method is repeated for each subgroup until all of the separations are not significant or the minimum node has been reached.

The conditional inference tree was obtained using the ctree() function in the Party package of the R language. Then, the conditional inference tree was used to classify the test set and the logarithmic probability of success was output. Finally, a cross table was used to compare the prediction with the actual situation.

### Random Forest (RF)

Random forest (RF) is a classifier that uses multiple trees to train and predict samples. Svetnik et al. generated multiple prediction models and summarized the results of the models to improve the classification accuracy in RF (13). The RF algorithm involves sampling sample units and variables to generate a large number of decision trees. From an intuitive point of view, each decision tree is a classifier (assuming that it is aimed at the classification problem); then, for an input sample, n trees will have n classification results. The RF integrates all of the voting results, and the category with the most voting times is designated as the final output.

Each tree is generated according to the following rules: 1. If the size of the training set is N, N training samples are randomly extracted from the training set and put back (this sampling method is called the bootstrap sample method) as the training set of the tree. The training set of each tree is different, and it contains repeated training samples. The training set of each tree and the final classification of trained tree are the same if there is no random sampling. If it is not sampling with return, the training samples of each tree are different and there will be no intersection. Therefore, each tree is “biased” and absolutely “one-sided”. 2. Every tree grows as much as possible, and there is no pruning process.

The RF() function in the RF package of R language was used to generate the RF. The RF package is a RF generated from a traditional decision tree. Then, the RF was used to classify the test set. The logarithmic probability of success was output. Finally, a cross table was used to compare the prediction with the actual situation.

### Algorithm Evaluation

After using each algorithm to train and test the data set, the performance of each algorithm is evaluated by different indicators, including specificity, sensitivity, positive/negative predictive rate, overall prediction accuracy and area under curve (AUC). The positive/negative predictive rate, which refers to the possibility of success/failure if the model predicts success/failure. The AUC reflects the accuracy of the algorithm.

### Statistical Analysis

Categorical variables were described as frequencies (percentages), and continuous variables were described as the mean ± standard deviation (SD). Pearson’s chi-square test and Student’s t-test were used for parametric comparisons. *P* value < 0.05 was considered to indicate statistical significance. All statistical analyses were performed using SPSS version 17 (SPSS Inc., Chicago, IL, USA).

### Human Ethics

This study was approved by the Medical Research Ethics Committee of The First Affiliated Hospital of USTC (Approve ID: 2021-RE-062).

## Results

### Clinical Data

We divided the female patients into the clinical pregnant and non-clinical pregnant groups. The clinical characters of the patients are summarized in **Table 1**. There was no significant difference between the two groups regarding some baseline characteristics (e.g., BMI, endometrial preparation protocol, number of transferred embryos per transferred cycle, number of good-quality embryos per transferred cycle, type of embryos transferred, serum estradiol level on the day of ET, serum progesterone level on the day of ET and endometrial morphology) except for age, EMT, GQR and type of infertility. Age was significantly lower in women who achieved a successful clinical pregnancy (30.7 *vs.* 32.7 years, *P* = 0.0002) compared to those who did not. The EMT of clinical pregnancy group was significantly thicker than that of non-clinical pregnancy group (9.5 *vs.* 9.0 mm, *P* = 0.001). The GQR and primary infertility rate in clinical pregnancy group were significantly higher than that in non-clinical pregnancy group (93.4% *vs.* 82.0%, *P* = 0.0001; 57.4% *vs.* 44.7%, *P* = 0.01), respectively.

### The Relationship Between the Outcome of ET and the Factors

**Figure 1:**
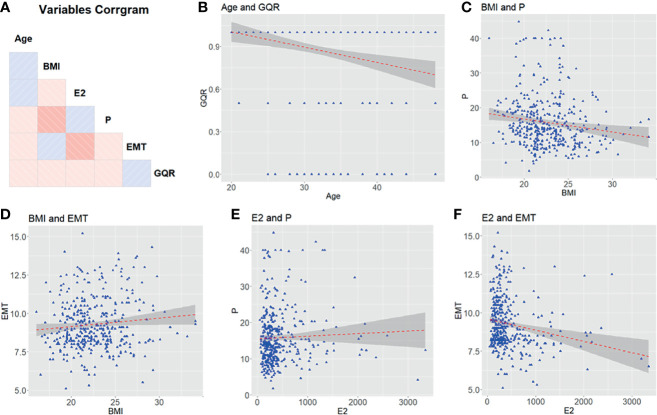
The relationship among clinical characters (age, BMI, E2, P, EMT and GQR) from patients undergoing embryo transfer. **(A)** Correlation matrix of 6 features. The blue and red squares show positive correlations and negative correlations, respectively. Color depth is positively correlated with correlation coefficient. **(B–F)** Comparison between features. BMI, body mass index; EMT, endometrial thickness on the day of progesterone treatment; GQR, good-quality embryo rate; E2, serum estradiol level on the day of embryo transfer; P, serum progesterone level on the day of embryo transfer.

**Figure 2:**
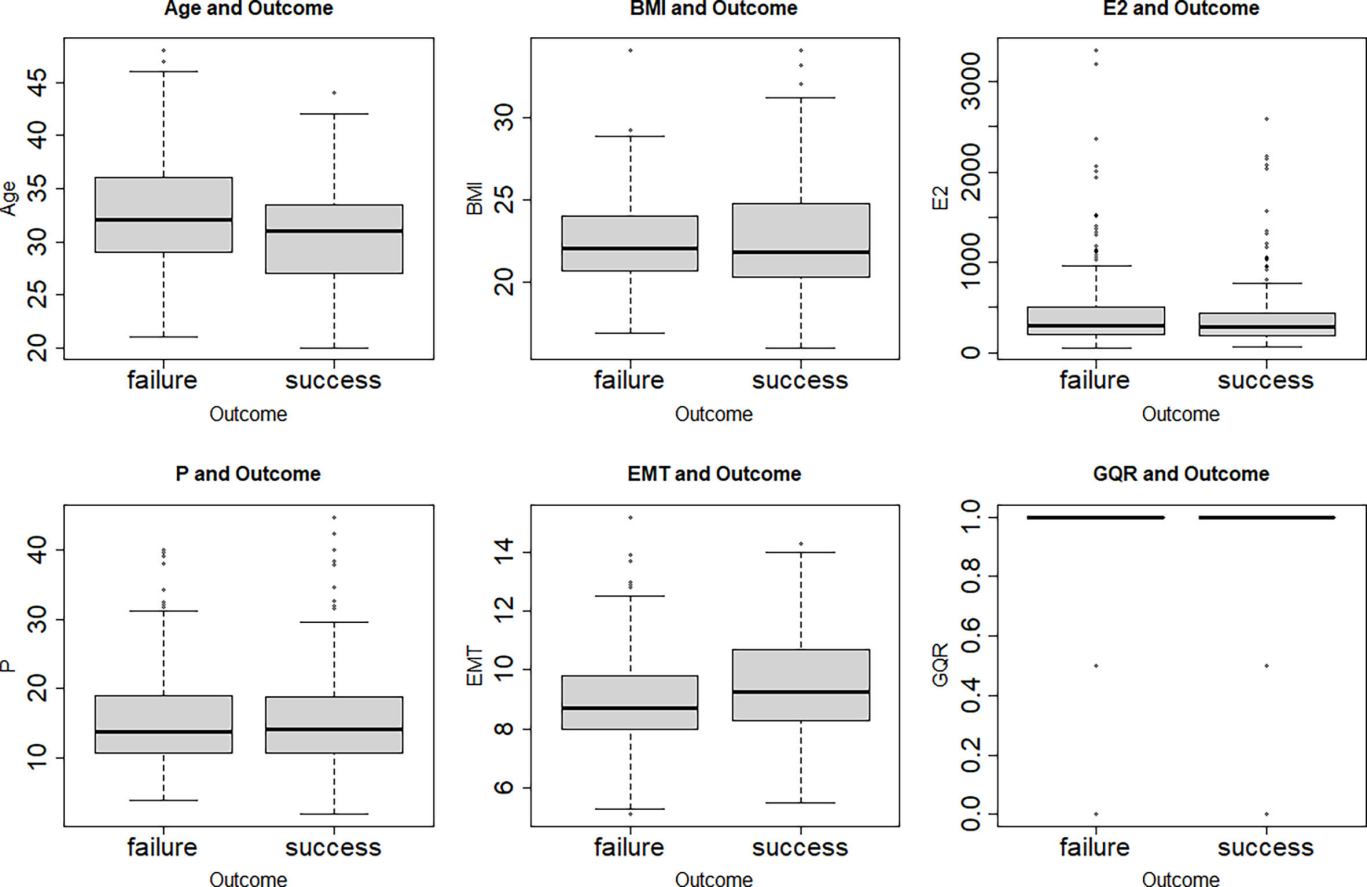
Box plots of the age, BMI, E2, P, EMT and GQR in clinical pregnancy (success) and non-clinical pregnancy (failure) group. There were significant differences between the two groups in age (t = 3.70, *P* = 0.0002), EMT (t = -3.26, *P* = 0.001) and GQR (t = -3.84, *P* = 0.0001). BMI, body mass index; EMT, endometrial thickness on the day of progesterone treatment; GQR, good-quality embryo rate; E2, serum estradiol level on the day of embryo transfer; P, serum progesterone level on the day of embryo transfer; success: success of clinical pregnancy; failure: failure of clinical pregnancy.

The clinical characters of 401 female patients who experienced hormone replacement FET cycles were analyzed in this study. The correlations among the characters are shown in **Figure 1**. No correlation was found between each two clinical characters (**Figure 1**). **Figure 2** showed that features were compared between the two groups. There were significant differences between the two groups in age, EMT and GQR (**Figure 2**).

### Evaluation of the Algorithms

**Figure 3:**
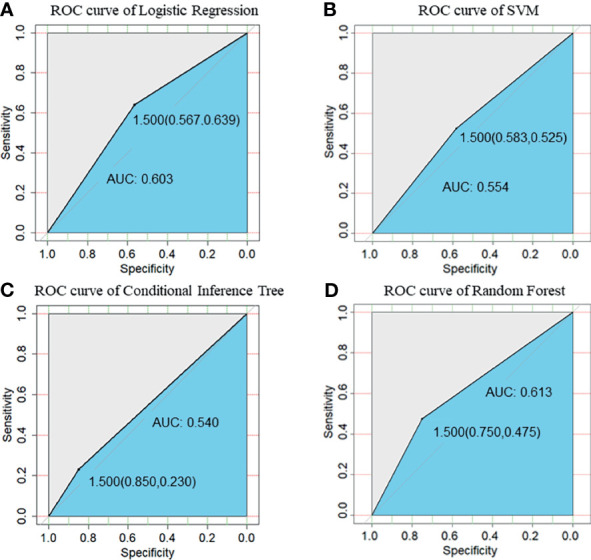
ROC curve of prediction of clinical pregnancy with clinical characters. **(A)** ROC curve of Logistic Regression; **(B)** ROC curve of SVM; **(C)** ROC curve of Conditional Inference Tree; **(D)** ROC curve of Random Forest. Clinical characters included age, body mass index, serum estrogen level on the ET day, serum progesterone level on the ET day, endometrial thickness on the day of progesterone treatment, good-quality embryo rate and type of infertility (primary or secondary). SVM, support vector machine.

**Figure 4:**
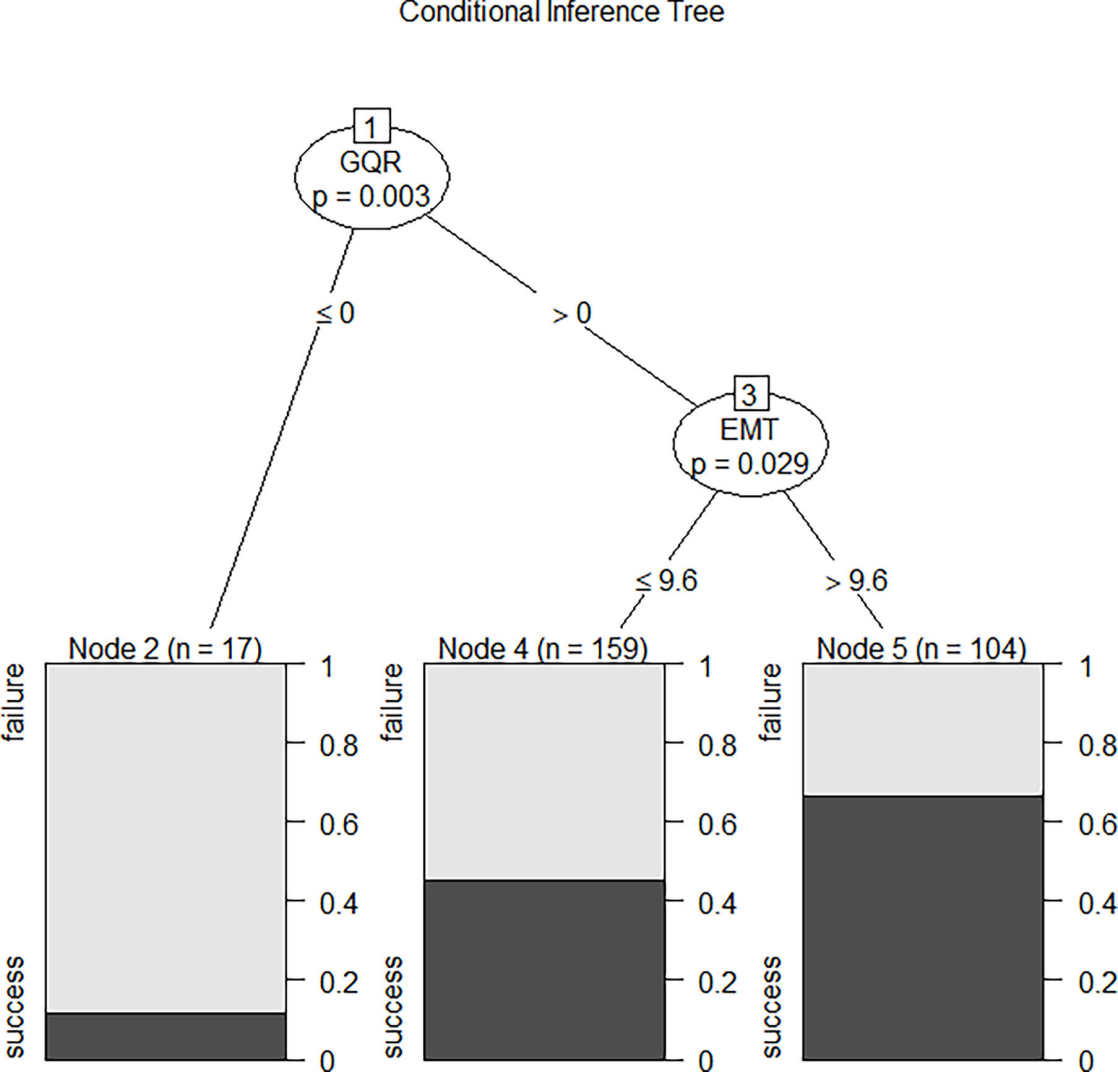
The conditional inference tree screened out two variables to predict the outcome of FET: the first was GQR, and the second was EMT. Among the patients with good-quality embryos, the clinical pregnancy (success) rate of FET was approximately 70% when EMT was thicker than 9.6 mm, but 50% when EMT was no thicker than 9.6 mm. For the patients without good-quality embryo, the clinical pregnancy (success) rate of FET was approximately 10%. EMT, endometrial thickness on the day of progesterone treatment; GQR, good-quality embryo rate; success, success of clinical pregnancy; failure, failure of clinical pregnancy.

We compared the performance of these four machine learning algorithms by using accuracy, sensitivity, specificity and positive/negative predictive rate (**Table 2**). Clinical characters includes age, BMI, E2, P, EMT, GQR and type of infertility. The predictive abilities of the four machine learning algorithms for clinical pregnancy were further analyzed with a receiver operating characteristic (ROC) curve (**Figure 3**). In addition, two variables were screen out through conditional inference tree to predict the results of FET: EMT and GQR. As shown in **Figure 4**, among the patients with good-quality embryos, the clinical pregnancy (success) rate of FET was approximately 70% when EMT was thicker than 9.6 mm, but 50% when EMT was no thicker than 9.6 mm. For the patients without good-quality embryo, the clinical pregnancy (success) rate of FET was approximately 10% (**Figure 4**).

**Table 2:** Performance comparison among the four machine learning algorithms in predicting clinical pregnancy.

Machine learning models	AUC	Sensitivity	Specificity	Positive predictive rate	Negative predictive rate	Accuracy
Logistic regression	0.603	0.64	0.57	0.60	0.61	0.60
Support vector machine	0.554	0.52	0.58	0.56	0.55	0.55
Conditional inference tree	0.540	0.30	0.80	0.60	0.53	0.55
Random forest	0.613	0.48	0.75	0.66	0.58	0.61

Predictive factors included age, body mass index, serum estrogen level on the day of embryo transfer, serum progesterone level on the day of embryo transfer, endometrial thickness on the day of progesterone treatment, good-quality embryo rate and type of infertility (primary or secondary).

## Discussion

The present study adopted machine learning to help doctors make correct predictions, which are beneficial for both doctors and patients. The results show that the machine learning algorithm is suitable for the prediction of pregnancy outcome after ET, and the conditional inference tree has better prediction ability and effective predictors. The advantages of this algorithm have also been verified in other medical disciplines (14). Patients without good-quality embryos can be advised to cancel transplantation to avoid unnecessary economic losses. With the increase in medical data, machine learning algorithms have been widely used in the rapid analysis of large amounts of data. Machine learning can predict clinical outcomes by defining data attributes and using clinical data and calculation algorithms (15). It can also improve the efficiency of predicting outcomes by building different algorithms for evaluation and comparison (16). Machine learning algorithms include traditional logistic regression, support vector machines, decision trees, random forests and so on. Goyal et al. established the machine learning model to predict a successful live-birth through 30 clinical features in IVF (15). Another study identified six classification models that can accurately predict early pregnancy loss (8). Moreover, Raef et al. used 82 attributes as predictive factors to predict ET outcomes with six dominant machine learning approaches (17). These studies indicated that RF model outpace other platforms in prediction. Currently, AI is intensively researched and widely used in IVF, especially in the selection of embryos (18). The purpose of using AI in IVF embryo selection is to eliminate the potential deviation of selection based on visual evaluation alone. VerMilyea et al. established the life whisper AI model which can improve the ability of predicting embryo viability compared with the traditional embryo grading method (19). A naïve Bayes model was established to predict the pregnancy outcome of individual embryos in an IVF cycle with the aim of providing decision support on the number of embryos transferred (20). In addition, the deep learning model was able to predict clinical pregnancy from time-lapse videos with an AUC of 0.93 [95% CI 0.92-0.94] (21). These studies demonstrated the potential of AI-based methods in improving the success rate of IVF laboratory. Furthermore, Yi et al. established a logistic algorithm and used FHR, GS, CRL, YSD and MA as features to predict the pregnancy outcome of 2601 ET samples (22). However, few studies have used machine learning algorithms to predict pregnancy outcomes during hormone replacement FET cycles (9, 23, 24).

In the process of FET, there are many factors affecting the pregnancy outcome, including the age, embryo quality, endometrial receptivity and so on. A previous study showed that clinical pregnancy outcomes after FET could be accurately predicted using objective parameters, such as the woman’s age (25). It has already been reported that the clinical pregnancy rate using blastocyst transfer decreases gradually with increasing maternal age, and the embryo implantation rate is significantly higher in women under the age of 35 (23, 26). In the four algorithms of the present study, age was not a strong predictor in the prediction of FET outcome. However, there were significant differences in age and GQR between clinical pregnancy group and non-clinical pregnancy group. Age may play an indirect role by affecting the quality of oocytes. It has been reported that age and embryo quality are independent factors affecting oocyte variability and embryo implantation potential (27–31). This finding is supported by the extensive evidence in the literature of an overall adverse effect of aging on oocyte quality and hence embryo quality (32, 33). Studies found that age was significantly different between the primary and secondary infertility groups (34). Primary infertility refers to patients who have never had a history of pregnancy. This conclusion is consistent with the clinical characteristics of patients included in this study.

Endometrial receptivity is regulated by many factors, such as the levels of serum estrogen and progesterone and the characteristics of the endometrium. In the hormone replacement cycle, exogenous progesterone is necessary for the transformation of the endometrium from the proliferative phase to the secretory phase in the hormone replacement cycle. Recent studies have shown that in the early luteal phase, there is a window period for the optimal progesterone level to permit embryo implantation (35). A premature increase in progesterone or failure to reach the threshold level can lead to early closure or nonopening of the implantation window, which eventually leads to embryo implantation failure.

Studies have reported the relationship between the hormonal level on the day of ET and the pregnancy outcome in FET cycles (36, 37). Yovich et al. used multiple comparison analysis, which showed that the likelihood of pregnancy in FET cycles under hormonal control is highly correlated with the circulating concentration of P (35). Boynukalin et al. also proposed the correlation between the progesterone level on the day of ET and the persistent pregnancy rate after a hormone replacement cycle (37–39). However, these transferred embryos were euploid blastocysts. The potential influence of age and embryo quality on pregnancy outcome was excluded. Niu et al. found that serum progesterone and estradiol on or before the day of ET did not predict pregnancy success in hormone replacement FET cycles (40, 41). The methods used in the above researches did not involve machine learning algorithms. However, we used machine learning algorithms to predict the pregnancy outcomes and concluded that serum E2 and P levels on the day of ET could not predict the pregnancy outcomes in hormone replacement FET cycles, suggesting that hormonal measurement on the day of ET in this method of endometrial preparation is unnecessary.

Richter et al. demonstrated that age, embryo quality, and EMT were related to clinical pregnancy (42). In addition to age, Michael et al. also identified EMT as an independent predictor of clinical pregnancy following blastocyst transfer. EMT thicker than 9.4 mm was identified as most predictive of a successful clinical pregnancy (24). In the present study, the EMT screened out by the conditional inference tree played important roles in predicting the pregnancy outcome, which is consistent with previous studies (4, 43, 44).

There are still some limitations in this study. The prediction accuracy of the present study is limited, which suggests that there may be some other attributes that can be included as follows: 1) stimulation approach 2) oocyte quality 3) time lapse based annotations 4) male factor infertility 4) culture conditions 5) ethnic variation. In addition, the clinical data used were derived from a single center of one hospital. Therefore, more data from other centers are needed to improve the prediction performance of the algorithm.

## Conclusion

Machine learning algorithms were used to establish four models to predict the pregnancy outcomes of patients preparing for FET with hormone replacement cycles. GQR and EMT were the predictors of clinical pregnancy screened out by conditional inference tree. This result will provide a reference for couples who are considering ET and help them make appropriate choices. This information might be useful in clinical practice because it showed there was no value in measuring E2 and P levels on the day of ET during hormone replacement cycles. In summary, these data demonstrated the potential for AI predictive models to contribute to IVF in the same way that they had contributed to other areas of human health. AI predictive models can reduce costs of assisted reproductive technology (ART) treatments by preventing repeated IVF cycles and high expenditures of ART cycles is one of the major barriers that have significant economic effects on communities especially in countries where public funding is used. In the future, we expect to improve the prediction performance of machine learning algorithms through the expansion of data sets and parameter types, hoping to find strong predictors that predict pregnancy outcomes accurately.

## Data Availability Statement

The original contributions presented in the study are included in the article/supplementary material. Further inquiries can be directed to the corresponding authors.

## Ethics Statement

This study was approved by the Medical Research Ethics Committee of The First Affiliated Hospital of USTC (Approve ID: 2021-RE-062). The patients/participants provided their written informed consent to participate in this study.

## Author Contributions

RL conceived and designed the experiments, performed the experiments, analyzed the data, prepared figures, authored or reviewed drafts of the paper, approved the final draft. SB and XJ authored or reviewed drafts of the paper. LL, XT, and SZ contributed reagents/materials/analysis tools. YW and BX conceived and designed the experiments, contributed reagents/materials/analysis tools, authored or reviewed drafts of the paper, approved the final draft. All authors contributed to the article and approved the submitted version.

## Funding

This study was supported by National Natural Science Foundation of China (82171599, 81971333 and 81901543), and the National Key Research and Development Project (2019YFA0802600). The funders had no role in study design, data collection and analysis, decision to publish, or preparation of the manuscript.

## Conflict of Interest

The authors declare that the research was conducted in the absence of any commercial or financial relationships that could be construed as a potential conflict of interest.

## Publisher’s Note

All claims expressed in this article are solely those of the authors and do not necessarily represent those of their affiliated organizations, or those of the publisher, the editors and the reviewers. Any product that may be evaluated in this article, or claim that may be made by its manufacturer, is not guaranteed or endorsed by the publisher.
